# PURSUING AGE-FRIENDLY UNIVERSITY PRINCIPLES AT A MAJOR UNIVERSITY: LESSONS IN GRASSROOTS ORGANIZING

**DOI:** 10.1093/geroni/igz038.2800

**Published:** 2019-11-08

**Authors:** Clare C Luz

**Affiliations:** Michigan State University, East Lansing, Michigan, United States

## Abstract

Rapid population aging presents opportunities for higher education to address major aging-related public issues facing society that have a direct impact on students, faculty, and both local and global communities. Students in virtually all disciplines will be working within the context of an aging society post-graduation and need to be prepared as they make career choices and enter the workforce. Further, faculty and staff are not only aging themselves but may be caregivers, which has an impact on health, income and productivity. Michigan State University (MSU) is now addressing these needs through a new program guided by Age-Friendly University (AFU) principles called AgeAlive that grew out of five years of grassroots organizing. Large-scale, research-intensive institutions present special challenges to pursuing AFU status but the lessons learned by AgeAlive may help any organization that wishes to become more age friendly. This session will review AgeAlive’s path to a recognized program with a clear vision, a strategic plan, two crosscutting goals including AFU designation, and five focus areas with initiatives in each area. Tools to help achieve these goals include an inventory of aging-related activity on campus and a virtual hub for networking and information exchange. Key steps in the program development process will be described as will recommendations related to choosing a model, cultivating champions, making decisions based on data, and building infrastructure. It will allow others to understand what challenges they may face and potential approaches to minimizing and overcoming these challenges in their own AFU journey.

